# Genetic Characterization of African Swine Fever Virus from Pig Farms in South Korea during Outbreaks in 2019–2021

**DOI:** 10.3390/v14122621

**Published:** 2022-11-24

**Authors:** Ki-Hyun Cho, Da-Young Kim, Min-Kyung Jang, Seong-Keun Hong, Ji-Hyoung Ryu, Hae-Eun Kang, Jee-Yong Park

**Affiliations:** Foreign Animal Disease Division, Animal and Plant Quarantine Agency, Gimcheon-si 39660, Republic of Korea

**Keywords:** African swine fever virus, pig farms, South Korea, virus isolation, genetic characterization

## Abstract

In South Korea, a total of 21 African swine fever (ASF) infected farms were confirmed during 2019–2021. ASF viruses (ASFVs) were isolated from the blood and spleen samples of the 21 affected farms and their genetic characteristics were analyzed. Phylogenetic analysis indicated that the 21 Korean ASFV strains belonged to p72 genotype II and serogroup 8. All isolates were of the intergenic region (IGR) II variant with 10 tandem repeat sequences between I73R and I329L and the central variable region (CVR) 1 variant of the B602L gene. There were no IGR variations between the A179L and A137R and between the MGF 505 9R and10R nor mutations in the O174L, K145R, MGF 505-5R, CP204L, and Bt/Sj regions. The genes of the 21 ASFV strains were identical to those of Georgia 2007/1 and Chinese and Vietnamese strains (Pig/HLJ/2018, China/2018/AnhuiXCGQ, and ASFV_NgheAn_2019); however, X69R of the J268L region of the 18th isolate (Korea/Pig/Goseong/2021) had three nucleotide (CTA) insertions at the 209th position, which led to the addition of one tyrosine (Y) at the C-terminal. This suggests that there are variations among ASFVs circulating in South Korea and the 18th ASFV-infected farm was due to a variant different from those of the other 20 pig farms.

## 1. Introduction

African swine fever (ASF) is one of the most fatal viral diseases affecting swine. ASF has high mortality and, due to the lack of effective vaccines and treatments, remains a huge threat to the pig industry and food security worldwide [1]. With recent advances in vaccinology, various attempts have been made to develop safe and effective vaccines for ASF. Several live attenuated vaccine candidates were shown to be promising, including virulence-associated gene deleted viruses, naturally attenuated viruses, and viruses attenuated by cell passages; however, several critical issues need to be resolved to commercialize the vaccine candidates, including safety, availability of stable cell lines, differentiating infected from vaccinated animals (DIVA), and cross-protection against different genotypes [2]. African swine fever (ASF) was first reported in Kenya in 1921 [3]. Until recently, ASF was endemic to sub-Saharan African countries and Sardinia in Italy. Since its introduction into Georgia in 2007, ASF has spread to Russia and Eastern Europe. In 2018, China reported its first ASF outbreak. The disease has since been transmitted to other neighboring Asian countries, including Vietnam, Myanmar, South Korea, the Philippines, Cambodia, Indonesia, Timor-Leste, Papua New Guinea, India, Bhutan, and Thailand (WOAH WAHIS Interface).

**Figure 1:**
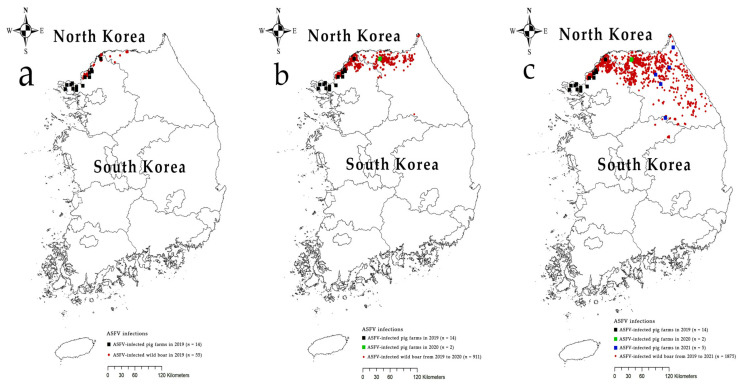
ASF outbreaks in domestic pig farms in South Korea during 2019–2021: (**a**) until 31 December 2019, (**b**) until 31 December 2020, and (**c**) until 31 December 2021. Black, green, and blue rectangles indicate ASFV-infected pig farms in 2019, 2020, and 2021, respectively. Red circles are the location where ASFV-infected wild boars were identified.

On 16 September 2019, the first ASF outbreak in South Korea was reported at a pig farm in Paju City, Gyeonggi Province. The index farm was located in the northwestern region near the border between South and North Korea. Until 9 October 2019, a total 14 ASF cases was confirmed in 4 cities/counties located in the northwestern border region [4]. On 3 October 2019, the first ASF-infected wild boar was identified in the northwestern demilitarized zone (DMZ) [5]. ASF has since expanded eastward and southward via the wild boar population. As of 31 October 2021, 1875 ASFV-infected wild boars were found in 4 and 17 cities/counties of Gyeonggi Province and Gangwon Province, respectively. This continued circulation and expansion of ASF indicates a significant risk of spillover to pig farms and poses a severe threat to the pig industry in South Korea. In October 2020, two cases were detected on pig farms in Gangwon Province. The infected premises were located in the north-central border region [6]. In May 2021, ASF was confirmed at one black pig farm in Yeongwol County, the southernmost municipality in Gangwon Province. In August 2021, one pig farm in Goseong County was confirmed to have an outbreak, followed by two further outbreaks in Inje and Hongcheon counties; additionally, one ASF case was detected at a pig farm in Inje County in October 2021 (Figure 1).

African swine fever virus (ASFV), the causative agent of ASF, is a large double-stranded DNA virus that is the only known member of the *Asfarviridae* family [7]. Depending on the isolates, the length of the ASFV genome ranges from 170 kb to 190 kb [8,9]. The ASFV genome encodes 150–200 proteins, including 50 structural proteins [10]. ASFV strains circulating worldwide have been classified into 24 different genotypes based on differences in the variable region of the p72 gene (B646L) [11,12]. ASFV can be subtyped using sequence information from the CD2v (EP402R), central variable region (CVR) of B602L, and tandem repeat sequence (TRS) region between the I73R and I329L genes. Analysis of other genes, such as O174L, K145R, MGF 505-5R, the CP204L encoding p30 protein, Bt/Sj region, J268L region, and IGRs between the A179L and A137R and between the MGF 505 9R and 10R region can be used to differentiate between closely related ASFV strains [13].

In a previous study [14], 14 ASFVs were isolated from positive samples from the infected pig farms during outbreaks in 2019, and several genes were analyzed. Their targets for genetic characterization included the p72 genotype, CD2v serogroup, CVR region of B602L, and IGR variation between the I73R and I329R and between MGF 505 9R and 10R. In this study, we isolated ASFV from seven infected pig farms between 2020–2021 and analyzed 12 genes from 21 ASFV strains in order to obtain information to discriminate between Korean ASFV isolates and possible source of the virus.

## 2. Materials and Methods

### 2.1. Virus Isolation

EDTA-treated whole blood or spleen samples were collected from 21 infected domestic pig farms between 2019–2021. Tissue samples were homogenized and centrifuged. The supernatant was filtered and inoculated into porcine alveolar macrophage (PAM) cells for virus isolation, according to the procedures of the Center for Animal Health Research, the European Union Reference Laboratory of ASF, and WOAH [15]. Briefly, PAM cells were seeded into 96-well tissue culture plates and incubated for 4 h at 37 °C in a CO_2_ incubator. The prepared samples were inoculated and 1% pig erythrocyte suspension was added. The virus in the cell supernatants was confirmed by OIE TaqMan quantitative polymerase chain reaction and the cyclic threshold (C_t_) was subsequently estimated. Virus isolation was confirmed via hemadsorption (HAD).

### 2.2. Fluorescent Antibody Test (FAT)

Virus antigens in PAM cells were also confirmed by fluorescent antibody test, according to the Diagnostic Manual of WOAH (2012). Four days after the first passage, the infected PAM cells were fixed in 80% acetone. Acetone was discarded and the plate was air-dried at room temperature. Anti-ASF VP72 monoclonal antibody (Eurofins Ingenasa, Madrid, Spain) was incubated for 1 h at 37 °C. After being washed with PBS three times, cells were incubated in goat anti-mouse IgG − h + I conjugated with FITC (Bethyl Laboratories, Montgomery, TX, USA). Fluorescence was observed and compared with mock infected PAM cells as a negative control.

### 2.3. PCR Assay

Nucleic acid was extracted from the collected samples using Maxwell^®^ RSC whole blood DNA kit for EDTA-treated whole blood and Maxwell^®^ RSC viral total nucleic acid purification kit (Promega, Madison, Wisconsin, USA) for tissue homogenates, processed through the Maxwell^®^ RSC 48 instrument, according to the manufacturer’s instructions. To genetically characterize the ASFV isolates, 12 gene markers were amplified and sequenced. First, we amplified the B646L gene encoding p72 for genotyping, and the EP402R encoding CD2v to classify serogroup, as previously reported [11,16,17]. To differentiate between Korean ASFV isolates, previously reported PCR primers and conditions were used to amplify CVR within the B602L gene [16], IGR between the I73R and I329L genes (IGR_I73R-I329L_) [18], IGR between the A179L and A137R (IGR_A179L-A137R_) [19], IGR between MGF 505 9R and 10R (IGR_MGF 505 9R/10R_) [14,20], CP204L encoding p30 [21], O174L, K145R, MGF 505-5R [22], Bt/Sj, and j286L regions [23]. The PCR products were submitted to Macrogen (Daejeon, Republic of Korea) for Sanger sequencing.

### 2.4. Analysis of the ASFV Isolates

All sequences of the 12 gene markers were analyzed using BioEdit 7.2 (Ibis Biosciences, https://www.mbio.ncsu.edu/bioedit/bioedit.html accessed on 15 November 2020). Phylogenetic analysis of nucleotide sequences of the p72 genotype and the EP402R gene for serogroup were conducted using MEGA X 10.2 software (http://www.megasoftware.net accessed on 19 November 2020)

## 3. Results and Discussion

### 3.1. Virus Isolation

**Figure 2:**
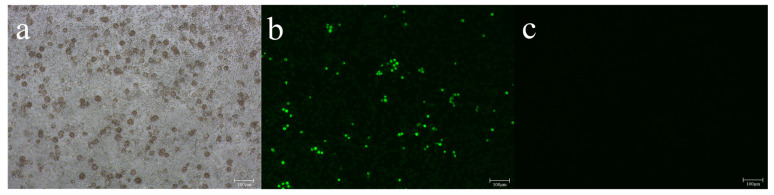
Confirmation of ASFV isolation (Korea/Pig/Yeongwol/2021) from spleens originating from the 17th infected farm. (**a**) HAD 6 days post inoculation of the first passage (×100) and (**b**) FAT 4 days post inoculation of the first passage (×100) compared with PAM cells infected with mock (**c**).

In addition to the 14 ASFVs previously isolated [14], 7 ASFV isolates were successfully isolated from the blood and spleen of infected pigs in farms from 2020 to 2021. The 16th isolate was confirmed after passaging for four times due to the low number of viral genomes with a C_t_ value of 34.3. All isolates showed the HAD phenomenon and were confirmed using the fluorescent antibody test (Figure 2). Information on the 21 isolates is shown in Table 1.

### 3.2. Genetic Characterization

**Figure 3:**
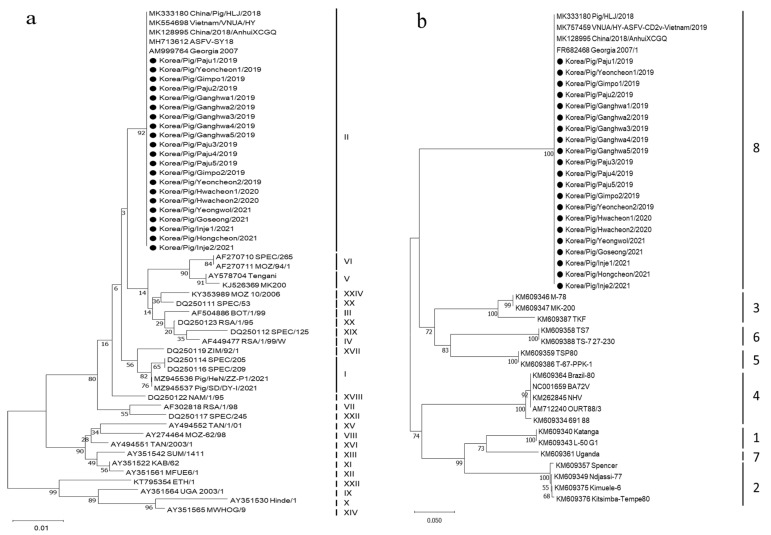
Phylogenetic trees of the 21 ASFV strains isolated from domestic pig farms in 2019–2021, constructed based on partial sequencing of the B646L and EP402R genes. (**a**) p72 genotype and (**b**) EP402R serogroup. The neighbor–joint method and Kimura 2-parameter model were used to construct both phylogenetic trees in MEGA X software (https://www.megasoftware.net/ (accessed on 15 November 2020)). Black dots indicate the Korean ASFV strains.

All 21 ASFV isolates were p72 genotype II, serogroup 8, CVR 1, and IGR_I73R-I329L_ II variant. The five genes of the first ASFV isolate in South Korea designated as Korea/Pig/Paju1/2019 have been deposited in GenBank with accession numbers MN603967 (B646L), MN603968 (E183L), MT335858 (EP402R), MN631140 (CVR), and MN603969 (IGR_I73R-I329L_); also, GenBank further contains the whole-genome sequence of Korea/Pig/Paju1/2019 (accession number MT748042). Phylogenetic trees of the p72 genotype and CD2v serogroups are shown in Figure 3.

The p72 genotype II is the most dominant ASFV genotype worldwide and ASFVs of this genotype have been detected in all Asian countries. It should be noted that recently p72 genotype I viruses have been reported to be circulating in China in addition to genotype II [24]. The EP402R encoding CD2v protein, which is involved in HAD, was used to classify serogroup [17]. Almost all p72 genotype II ASFV strains isolated from Europe and Asia belonged to serogroup 8.

To obtain an enhanced resolution of the genetic characteristics of the ASFV isolates, 10 additional genes were analyzed. The CVR sequence of B602L was used to increase the intragenotypic discrimination. The CVR region is translated into amino acid sequence and grouped together. Overall, the 21 Korean isolates had a tandem amino acid repeat sequence (BNDBNDBNAA) within the CVR region, which corresponded to the p72 genotype II CVR1 (GII-CVR1, Georgia variant type). The GII-CVR1 profile was 100% identical to Georgia 2007/1, Pig/HLJ/2018, China/2018/AnhuiXCGQ, and VNUA/HY-ASF1. In Estonia, GII-CVR1 variants with single nucleotide polymorphism (SNP) 1 and GII-CVR2 deleted with 3 amino acid tetramer repeats (CASMCADTNVDT) were detected [25].

IGR between I73R and I329L (IGR_I73R-I329L_) is a genetic marker that can further discriminate between p72 genotype II viruses. Currently, four IGR_I73R-I329L_ variants have been identified: IGR_I73R-I329L_ I, IGR_I73R-I329L_ II with 10 nucleotides (nt) of TRS in comparison to IGR_I73R-I329L_ I, IGR_I73R-I329L_ III containing additional 10 nt of TRS in comparison to IGR_I73R-I329L_ II, and IGR_I73R-I329L_ IV possessing extra 10 nt of TRS in comparison to IGR_I73R-I329L_ III. The p72 genotype II IGR_I73R-I329L_ II is the most frequently identified in type in Europe and Asia. IGR_I73R-I329L_ I, III, and IV can be formed owing to the continuous circulation of ASFV in a pig population of a region. In South Korea, all ASFVs isolates from domestic pig farms in South Korea during 2019–2021 were IGR_I73R-I329L_ II, while IGR _I73R-I329L_ I, II, and III were detected in the wild boar population during surveillance in the northwestern border region in 2019 [26]. In China, IGR_I73R-I329L_ I, II, and III were found from wild boar in the northeastern border region (Jilin province) in 2018 and pig farms in the southern region in 2019, respectively [27,28]. IGR _I73R-I329L_ I, II, and III were also reported in Vietnam [29]. In Poland, where ASF has been endemic since 2014, IGR _I73R-I329L_ I, II, III, and IV were detected [22].

The IGRs between the A179L and A137R (IGR_A179L-A137R_) and between the MGF 505 9R and 10R (IGR_MGF 505 9R/10R_) are genetic markers used for subgrouping p72 genotype II viruses [19]. Two ASFV isolates with 11 nt (GATACAATTGT) of TRS in IGR_A179L-A137R_ were found in southern Vietnam. One strain (ASFV/VN/Pig/Hanoi/02) was IGR_I73R-I329L_ II, while the other (ASFV/VN/Pig/Hanoi/07) IGR_I73R-I329L_ I [30]. Three IGR_MGF 505 9R/10R_ variant into three types: MGF-1 without TRS insertion, MGF-2 with 17 nt of TRS (GATAGTAGTTCAGTTAA), and MGF-3 containing an additional 17 nt of TRS compared to MGF-2. MGF-2 was previously confirmed from seven Russian strains and nine Polish isolates [20,31]. A Russian ASFV isolate (Tver1112/Zavi) was identified as MGF-3. None of the 21 Korean ASFVs contained TRS sequences in IGR_A179L-A137R_ and IGR_MGF 505 9R/10R_.

In Eastern Europe, three genes, O174L, K145R, and MGF 505-5R, were used as molecular fingerprints to enhance intragenotypic resolution. In Poland, in-depth insight into the spatiotemporal clustering of ASFVs was obtained based on the analysis of these genes with IGR_I73R-I329L_ type. There are three types of O174L variants: variant I, identical to that of Georgia 2007/1; variant I, with SNP; and variant II, with inserts of 14 nt length TRS (CAGTAGTGATTTT). For K145R and MGF 505-5R, variants were divided into two groups. Variant I was 100% homologous to those of Georgia 2007/1, while variant II contained SNPs specific to Polish and Ukrainian isolates, which correspond to C65167A in K145R and G38332A in MGF 505-5R, respectively [22]. None of the 21 Korean isolates analyzed here possessed variations in the three genes.

**Figure 4:**
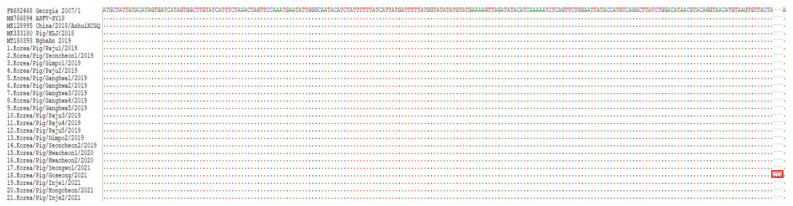
Analysis of X69R of the J286L region in ASFV strains isolated in pig farms in South Korea during 2019–2021. The 18th isolate, designated as Korea/Pig/Goseong/2021, contains three nucleotide (CTA) insertion on the 209th position of X69R in the J286L region (red rectangle), leading to the addition of one tyrosine (Y).

The CP204L gene encoding the p30 and Bt/Sj region of the 21 Korean ASFV strains was identical to those of Georgia 2007/1, with no mutations. The J268L region located in the left variable region included ASF G ACD 00320, 00330, 00350, and X69R genes at 19436–20581 positions in the Georgia 2007/1 strain. There was no variation in ASF G ACD 00320, 00330, and 00350 genes in Korean ASFV strains, which were identical to those of Georgia 2007/1; however, X69R gene of the 18th isolate, Korea/Pig/Goseong/2021, possessed 3 nt (CTA) insertion at the 209th position, leading to the addition of one tyrosine (Y) at the C-terminal (Figure 4).

All 21 ASFV strains isolated from pig farms in South Korea during the 2019–2021 outbreaks belonged to p72 genotype II, serogroup 8 with IGR _I73R-I329L_ II, and CVR1. They did not contain TRS insertions in IGR_A179L-A137R_ and IGR_MGF 505 9R/10R;_ in addition, no variations in the O174L, K145R, MGF 505-5R, CP204L, and Bt/Sj regions were found among the 21 Korean isolates. The analyzed genes were identical to those of Georgia 2007/1, Chinese strains Pig/HLJ/2018 and China/2018/AnhuiXCGQ, and Vietnamese strain ASFV_NgheAn_2019; however, the X69R gene of the J268L region in the 18th isolate (Korea/Pig/Goseong/2021) possessed one tyrosine (Y) insertion at the 209th position. This suggests that there are slight variations of ASFVs circulating in South Korea and the source of the virus of 18th ASF-infected farm was due to a variant different from that in the other 20 pig farms.

Analysis of whole-genome sequence of ASFV isolates can fully characterize the isolates. Though whole-genome sequencing itself can be over quickly by next-generation sequencing, subsequent genetic analysis is highly time-consuming. On the other hand, investigation of select gene markers of ASFV isolates by Sanger sequencing has important merit as it can be performed quicker to identify significant variations between ASFV isolates and allows for the rapid accumulation and sharing of data for specific genes, before the whole-genome sequence is eventually shared. To provide more comprehensive data on the viral genome, further studies are required to investigate other possible differences between isolates through analysis of other potential gene markers and whole-genome sequencing.

## Figures and Tables

**Table 1:** Summary of the isolation and genetic characterization results from 21 ASFV isolates from pig farms in South Korea during the 2019–2021 outbreaks.

	Isolate Name	Organ of Origin	qPCR C_t_	HAD	p72 Genotype	CD2v Serogroup	IGR_I73R-I329L_	CVR	IGR_MGF 505 9R/10R_	IGR_A179L-A137R_	O174L	K145R	MGF 505-5R
1	Korea/Pig/Paju1/2019	Spleen	17.1	Positive	II	8	II	CVR1	MGF-1	No TRS	I	I	I
2	Korea/Pig/Yeoncheon1/2019	Spleen	17.2	Positive	II	8	II	CVR1	MGF-1	No TRS	I	I	I
3	Korea/Pig/Gimpo1/2019	Blood	15.4	Positive	II	8	II	CVR1	MGF-1	No TRS	I	I	I
4	Korea/Pig/Paju2/2019	Spleen	15.3	Positive	II	8	II	CVR1	MGF-1	No TRS	I	I	I
5	Korea/Pig/Ganghwa1/2019	Blood	13.3	Positive	II	8	II	CVR1	MGF-1	No TRS	I	I	I
6	Korea/Pig/Ganghwa2/2019	Blood	15.4	Positive	II	8	II	CVR1	MGF-1	No TRS	I	I	I
7	Korea/Pig/Ganghwa3/2019	Blood	15.5	Positive	II	8	II	CVR1	MGF-1	No TRS	I	I	I
8	Korea/Pig/Ganghwa4/2019	Blood	16.0	Positive	II	8	II	CVR1	MGF-1	No TRS	I	I	I
9	Korea/Pig/Ganghwa5/2019	Spleen	17.6	Positive	II	8	II	CVR1	MGF-1	No TRS	I	I	I
10	Korea/Pig/Paju3/2019	Spleen	18.1	Positive	II	8	II	CVR1	MGF-1	No TRS	I	I	I
11	Korea/Pig/Paju4/2019	Blood	15.4	Positive	II	8	II	CVR1	MGF-1	No TRS	I	I	I
12	Korea/Pig/Paju5/2019	Spleen	16.4	Positive	II	8	II	CVR1	MGF-1	No TRS	I	I	I
13	Korea/Pig/Gimpo2/2019	Spleen	18.1	Positive	II	8	II	CVR1	MGF-1	No TRS	I	I	I
14	Korea/Pig/Yeoncheon2/2019	Blood	15.5	Positive	II	8	II	CVR1	MGF-1	No TRS	I	I	I
15	Korea/Pig/Hwacheon1/2020	Spleen	17.2	Positive	II	8	II	CVR1	MGF-1	No TRS	I	I	I
16	Korea/Pig/Hwacheon2/2020	Blood	34.3	Positive	II	8	II	CVR1	MGF-1	No TRS	I	I	I
17	Korea/Pig/Yeongwol/2021	Spleen	17.3	Positive	II	8	II	CVR1	MGF-1	No TRS	I	I	I
18	Korea/Pig/Goseong/2021	Spleen	17.1	Positive	II	8	II	CVR1	MGF-1	No TRS	I	I	I
19	Korea/Pig/Inje1/2021	Blood	13.2	Positive	II	8	II	CVR1	MGF-1	No TRS	I	I	I
20	Korea/Pig/Hongcheon/2021	Blood	25.7	Positive	II	8	II	CVR1	MGF-1	No TRS	I	I	I
21	Korea/Pig/Inje2/2021	Spleen	17.7	Positive	II	8	II	CVR1	MGF-1	No TRS	I	I	I

## Data Availability

The data presented in this study are available on request from the corresponding author. The original contributions generated for the study are included in the article. Further inquiries can be directed to the corresponding author.
